# Parenting and adolescent suicidal behavior

**DOI:** 10.1016/j.eclinm.2021.100728

**Published:** 2021-01-28

**Authors:** Rahul Shidhaye

**Affiliations:** Senior Research Scientist and Associate Professor of Psychiatry, Pravara Institute of Medical Sciences, Loni, India

Adolescence is a critical and challenging period during human development and globally every third death due to suicide occurs in young people [1]. This important public health issue is the focus of the study by Sayedul Ashraf Kushal and colleagues, published in *EClinicalMedicine* [2]. The authors present data from the Global School-based Health Survey (GSHS) and explore the association between parent-adolescent relationship and adolescent suicidal behaviors in 52 countries across different regions of the world. A quarter of the adolescents aged 12–15 years (26.2% girls and 23% boys) reported suicidal behavior (suicidal ideation/suicide planning/suicide attempt). Perception by the adolescents that parents understood their problems, monitored academic and leisure time activities, and respect of their privacy, were found to have a protective effect on suicidal behaviors. The positive influence of parent-adolescent relationship on adolescent suicidal behavior was slightly stronger in girls than boys. In low-income countries compared to lower-middle, upper-middle, and high-income countries, parent-adolescent relationship had lesser influence on adolescent suicidal behavior.

The impact of parents-adolescent relationship on adolescent suicidal behavior is still an understudied area of research and much of the evidence comes from high-income countries. In this context, the focus of this study on parent-adolescent relationship as a primary explanatory variable for adolescent suicidal behavior is a novel and awaited step. The findings of the study are in agreement with a previous study published in *EClinicalMedicine* which reported protective effects of parental understanding and monitoring and negative effects of parental control on anxiety and suicidal ideation in adolescents [3]. Similarly, a study based on the National Survey on Drug Use and Health (in the United States) also found that parental involvement especially in monitoring the home-work and related academic activities had positive effect on adolescents, especially 12–13 years old [4]. However, an analysis of GSHS data from 2003–2015 involving adolescents aged 13–17 years did not find any association of parental factors with suicidal ideation and/or suicidal attempts [5]. Another study using GSHS data found that parental over-protection may lead to increased suicide risk especially in boys from low-and-middle income countries [6]. These differences can be possibly explained by difference in the use of data from various phases of the GSHS and variable inclusion of countries in the analysis.

Suicide and suicidal behavior are complex phenomena, and a range of individual, population, and environmental factors are known to increase suicide risk [7]. Parent-adolescent relationship is one of the multiple factors that can have an influence and it is quite challenging to use it as a measurable ‘variable’ as well as quantify its impact on suicide risk in adolescents. A recent systematic review noted considerable variation in conceptualization of various parenting styles and practices as around 40 different instruments (some standardized and some ad-hoc) have been used to measure this variable [8]. It is important to consider that the GSHS only includes few questions related to parenting as the broader purpose of the survey is to collect data on health behaviors and protective factors to support school health and youth health programs. Additional caution is necessary while interpreting these results due to the cross-sectional design of the GSHS study and the self-reported nature of the data. Further, although GSHS is a representative population-based study, it only includes school-going adolescents. Thus, it is difficult to comment on school dropouts, especially adolescent girls in low-and-middle income countries who may experience higher vulnerability. In addition, the analysis in the study by Kushal et al. [2] includes data from 52 countries with no representation from populous countries such as India, China, Pakistan, Brazil, and South Africa.

Cultural, religious, and community level factors influence parenting practices and parent-adolescent relationship. The study highlights important regional differences, especially those related to ‘respecting privacy’ in a South Asian context and no association of any parental factors with adolescent suicidal behavior in African countries. It is quite likely that adolescents across different cultures understand and interpret concepts like ‘privacy’ and ‘monitoring’ differently and that socioeconomic factors may influence suicide risk in adolescents, particularly in low-resource settings.

Despite large variations across regions and countries, this study underlines the importance of parental factors in influencing mental health and suicide risk in adolescents. Emerging evidence from literature suggests that family-focused interventions have beneficial effects in improving mental health of children and adolescents [9]. Parenting practices are largely influenced by traditional values and social norms. The advent of globalization and rapid evolution of cultures have posed new challenges for both parents and adolescents. A recent report by the UNICEF titled ‘Parenting Adolescents’ recommends systematic and integrated support to improve parental skills [10]. Building on the findings of this study (and other similar), the focus of the future research should be to design, implement, and evaluate culturally appropriate interventions to improve parental relationship as a target for suicide prevention. These interventions should specifically focus on strengthening parental competencies to help adolescents cope with the developmental challenges and attain mental well-being.

## Author contributions

Rahul Shidhaye: Conceptualization, literature search, writing-original draft, finalization of the draft.

## Declaration of Competing Interest

Dr. Shidhaye has nothing to disclose.
